# Nanoporous Membranes for the Filtration of Proteins from Biological Fluids: Biocompatibility Tests on Cell Cultures and Suggested Applications for the Treatment of Alzheimer’s Disease

**DOI:** 10.3390/jcm11195846

**Published:** 2022-10-01

**Authors:** Thomas Gabriel Schreiner, Bogdan Ionel Tamba, Cosmin Teodor Mihai, Adam Lőrinczi, Mihaela Baibarac, Romeo Cristian Ciobanu, Bogdan Ovidiu Popescu

**Affiliations:** 1 Faculty of Medicine, University of Medicine and Pharmacy “Carol Davila”, 050474 Bucharest, Romania; 2 Department of Neurology, University of Medicine and Pharmacy “Gr. T. Popa”, 700115 Iasi, Romania; 3 Department of Electrical Measurements and Materials, Faculty of Electrical Engineering and Information Technology, Gheorghe Asachi Technical University of Iasi, 700050 Iasi, Romania; 4 Advanced Research and Development Center for Experimental Medicine (CEMEX), University of Medicine and Pharmacy “Gr. T. Popa”, 700155 Iasi, Romania; 5 National Institute of Materials Physics, 405A Atomistilor St., 077125 Măgurele, Romania; 6 Neurology Department, Colentina Clinical Hospital, 020125 Bucharest, Romania; 7 Laboratory of Cell Biology, Neurosciences and Experimental Myology, ‘Victor Babes’ National Institute of Pathology, 050096 Bucharest, Romania

**Keywords:** Alzheimer’s disease, amyloid-beta, nano-membrane, protein filtration, biocompatibility, functionalization, atomic layer deposition

## Abstract

Background: Alzheimer’s disease has a significant epidemiological and socioeconomic impact, and, unfortunately, the extensive research focused on potential curative therapies has not yet proven to be successful. However, in recent years, important steps have been made in the development and functionalization of nanoporous alumina membranes, which might be of great interest for medical use, including the treatment of neurodegenerative diseases. In this context, the aim of this article is to present the synthesis and biocompatibility testing of a special filtrating nano-membrane, which is planned to be used in an experimental device for Alzheimer’s disease treatment. Methods: Firstly, the alumina nanoporous membrane was synthesized via the two-step anodizing process in oxalic acid-based electrolytes and functionalized via the atomic layer deposition technique. Subsequently, quality control tests (spectrophotometry and potential measurements), toxicity, and biocompatibility tests (cell viability assays) were conducted. Results: The proposed alumina nanoporous membrane proved to be efficient for amyloid-beta filtration according to the permeability studies conducted for 72 h. The proposed membrane has proven to be fully compatible with the tested cell cultures. Conclusions: The proposed alumina nanoporous membrane model is safe and could be incorporated into implantable devices for further in vivo experiments and might be an efficient therapeutic approach for Alzheimer’s disease.

## 1. Introduction

Efficient chemical separations of bio-organic substances, based on the availability of reliable multifunctional membrane materials with well-defined nanoscale properties, are essential for medical diagnostic and therapeutic applications [1]. For most purposes, the economy of the membrane is closely related to its transport properties: permeability, which determines the productivity of separation, and selectivity, which determines the efficiency of separation [2]. Although ideal membranes are both very selective and very permeable, in reality, there is always a trade-off between these two parameters. To overcome this inherent trade-off, a rational design of the membrane must combine a selection of the most suitable materials (with respect to fluid–solid surface interactions), the optimal microstructure characteristics (pore size, porous volume, pore distribution, connectivity, and tortuosity), and the most favorable architecture (surface-to-volume ratio, hydrodynamics) [3]. Reactive and non-reactive chemicals occurring at solid–fluid interfaces play a key role in membrane performance, influencing its permeability (i.e., permeability ratio of a single component), selectivity, and sustainability (prevention of clogging and long-term stability).

Separation and purification processes are performed by allowing species/molecules to pass through the membrane as a result of a driving force. Most transport processes take place due to a difference in chemical potential (with pressure and concentration as the main contributors) [4].

Membranes can be manufactured with either symmetrical or asymmetrical structures, and with different configurations (flat sheets, tubes, honeycombs) [5]. Porous membranes can be classified according to their pore size, with different pore sizes suitable for specific applications (see Table 1) [6,7]. While macropores allow the filtration of oily emulsions and bacteria, mesopores separate most viruses, and micropores can be used to separate antibiotics, ions, and small neutral molecules (either in the gas phase or in the liquid phase).

Nanoporous materials obtained by electrochemical anodizing techniques are ideal for nanomembrane development, given the high porosity and narrow pore size distributions (pore diameters up to ~10 nm and extended over large regions of a few square centimeters) [8]. These high porosity values and narrow pore size distributions are not available through other nanofabrication technological approaches, such as ultraviolet (UV) or e-beam lithography techniques. In addition, proper control and modeling of electrochemical anodizing parameters (voltage, current density, and temperature) allow for the simple design of complex pore morphologies, such as asymmetric or funnel-shaped nanopores [9]. Moreover, anodic nanoporous oxides have high chemical and thermal stability, and good biocompatibility, which can be further improved by surface functionalization techniques [10]. Among the various possibilities, atomic layer deposition (ALD) stands out for its ability to deposit compliant coatings on 3D nanostructured substrates, with outstanding thickness and uniformity control [11]. Various biocompatible materials, such as silicon dioxide (SiO_2_) or titanium dioxide (TiO_2_), can be easily deposited by this technique to a thickness of a few nanometers [12]. These functional coatings allow for the modification of the pore size and geometry (cone-shaped pores) and also improve the biocompatibility and pre-selection of diffusive transport properties [13]. Finally, it is desirable to cover the surface with specific materials to reduce the biofouling effect of nanoporous membranes, with various techniques such as plasma jet, laser topological covering with hydrophilic/charged/polyethylene glycol networks, and the hydrophobic carbon coating currently available [14].

Scalability for industrial production must consider technical–economic aspects and resource consumption along with the minimization of design costs. In this context, nanoporous alumina membranes, obtained via the cost-effective and precisely adjusted two-stage anodizing method, are optimal in order to obtain inorganic filter membranes with almost monodisperse values of pore diameter in the range of 8–10 nm [15]. Electrochemical anodizing of high purity aluminum is performed at controlled temperature, basic pH, and applied anodic voltage. The ALD technique is subsequently performed to coat the open surface of the nanoporous alumina membranes in an ultra-thin (2–4 nm) layer of chemically resistant material (e.g., SiO_2_; TiO_2_). This layer serves two purposes: (i) it improves the biocompatibility, biostability, and chemical resistance of the membranes; and (ii) it allows for the precise control of the final pore diameter, allowing its reduction from 15 nm to the desired target size (8–10 nm) [16].

Via the two-stage anodizing method, nanoporous alumina membranes can be obtained at an industrial scale starting from aluminum foils, then undergoing subsequent changes such as the obtaining of very orderly pore networks with a honeycomb structure, with pore radii usually ranging from 10 nm to 50 nm and thickness between 10 μm and 100 μm. The well-defined porous structure and the possibility of changing both the pore size and the nature of the surface via the ALD procedure allow their application in controlled molecular release devices [17]. In these applications, the nanoporous alumina membranes are in contact with solutions containing molecules (neutral, charged, and even ions), which flow along the pores of the membrane due to the concentration gradients.

Industrial quality control of nanoporous alumina membranes should involve microstructural and chemical characterization. The differences between the diffusive parameters determined for the different samples could provide information on the influence of the interfacial effects on the diffusive transport of the charged species. To assess chemical stability and durability, high-resolution scanning electron microscopy (HR-SEM) and high-resolution transmission electron microscopy (HR-TEM) techniques will be used to characterize the microstructure/nanostructure of nanoporous membranes produced and coated with ALD. The chemical characterization should be performed via energy-dispersive X-ray spectroscopy (EDX) and X-ray photoemission spectroscopy (XPS) techniques [18]. In addition, the in vitro corrosion test should be performed to study the durability, paying attention to the chemical stability of the samples exposed to conditions similar to those of the test.

With recent advancements in the research and industry fields related to nanostructures, these promising perspectives should also be translated into clinical practice. The neurological domain is one of the important targets, as central nervous system (CNS) disorders are becoming increasingly widespread [19], with devastating consequences for patients and their relatives. Neurodegenerative diseases, Alzheimer’s disease (AD) in particular, have been the focus of many researchers due to their significant socioeconomic impacts. According to epidemiological data, AD affects more than 5 million people in the United States, and its prevalence is estimated to triple by 2050 [20]. Moreover, early diagnosis is difficult, while in the late stages, patients are totally dependent on external care [21]. 

In addition, therapeutic strategies are limited and mostly inefficient because of the complexity of the brain, particularly due to the poor permeability of the blood–brain barrier (BBB). Research on this topic has shown that only 5–10% of drugs are able to pass the BBB because of their large size and low-fat solubility [22]. The lack of effective technologies for the delivery of medicines through the BBB prevents the development of effective therapies for CNS diseases. Among the strategies explored to boost the passage of pharmacological agents through the BBB, the most direct is intraparenchymal delivery, as well as the intrathecal release into the cerebrospinal fluid (CSF) [23,24]. However, the administration of drugs directly into the brain tissue or CSF is associated with important side effects due to the direct impact of the drugs on the cells. Or, there is also the possibility that the drug passes quickly into the bloodstream, with minimal penetration of brain parenchyma.

The current therapeutic approaches mainly rely on cholinesterase inhibition [25], thus increasing the concentration of acetylcholine in certain parts of the brain, and by inhibition of N-methyl-D-aspartate (NMDA) receptors [26], preventing glutamate action, glutamate being a neurotransmitter that leads to neuronal excitability and excessive stimulation in AD. 

To date, various therapeutic agents have been investigated to develop a disease-modifying treatment: enzyme inhibitors to prevent plaque formation [27], monoclonal antibodies to remove plaques already formed [28], vaccination against amyloid plaques [29], and combinations of anti-inflammatory drugs [30]. Unfortunately, most of the studied therapeutic agents have not entered daily clinical practice as a result of inefficacy and significant adverse effects. We envision an alternative approach based on an implantable device comprising a selective membrane developed via nanotechnology as an essential component that may ensure the removal of amyloid beta (Aβ) directly from the CSF. 

In the context of multiple failures of the previous anti-dementia therapeutic approaches, this article aims to present a potential solution for anti-AD therapies within the manufacture of a novel nanoporous membrane to be incorporated in implantable devices for Aβ filtration.

## 2. Materials and Methods

### 2.1. Theoretical Starting Point—Basic Concepts

Nanoporous structures consisting of straight parallel channels with well-defined but highly modifiable lattice parameters (nanopore size and length, membrane porosity) have become of increasing interest recently due to their growth in applications such as nanofiltration, biosensors, and nanofluidic devices [31]. Nanoporous alumina membranes (NPAM) with a one-dimensional (1D) spatially distributed and vertically aligned channel structure have attracted a great deal of interest in the development of monodisperse and self-ordered 1D nanostructures. By using a growth-limiting template, such as a solid-state acid catalyst, for the matrix of ceramic materials, in the creation of NPAMs, mass transfer efficiency is improved, and the distribution of the active components on the matrix is uniform, with subsequent excessive local thermal reactions and a reduction of by-products generation. Although efficient technology for separating neutral molecules via nanoporous membranes is mainly dependent on the membrane’s pore size, surface loading can also have a significant effect on the transport or concentration of biomolecules and ions. 

Among the various techniques used to manufacture membranes with a well-defined nanostructure, polymeric membranes with etched channels and NPAMs obtained by the two-stage anodizing method of aluminum are the most commonly used. In both cases, porosity and pore radius depend on the preparation and post-treatment conditions. However, narrow nanopores and highly selective membranes can be obtained by different types of surface modifications and membrane functionalization. In particular, the atomic layer deposition (ALD) technique has been used to properly adjust the pore size of membranes, as well as to modify the physicochemical characteristics of the surface, such as charge or hydrophilicity, which in turn can be of great interest in the filtration/diffusion of aqueous solutions, as they may affect the selectivity of the membranes.

The ALD technique is based on the vapor deposition method, but with great potential for the production of very thin and compliant oxide layers, achieving optimal control of their thickness and composition on an atomic scale. Based on sequential and self-limiting reactions, ALD offers exceptional deposition compliance on successive structures, with a high length-to-diameter ratio and a size-adjustable thin film composition. Consequently, ALD is a remarkable technique for surface modification with a homogeneous oxide layer on the inner pore walls of nanoporous membranes. However, due to the high-temperature values required for some of the reactions, ALD is mainly used for functionalizing and modifying the surfaces of ceramic membranes, and only for adapting the size of polymer pores (in the case of polycarbonate membranes).

Via the ALD technique and using oxalic acid solutions (Ox NPAM sample), metal oxides are deposited on the surface of the NPAMs. A wide range of simple metal oxide layers (Al_2_O_3_, SiO_2_, TiO_2_, Fe_2_O_3_, and ZnO) can be deposited, as well as combinations of two-layer structures, the first layer consisting of SiO_2_ and the second layer consisting of Al_2_O_3_ or aluminum zinc oxide (AZO). Morphological and chemical changes in the pores of the Ox NPAM sample associated with ALD functionalization treatment can be analyzed by scanning electron microscopy (SEM) and X-ray spectroscopy with local energy dispersion (EDS).

It is thus possible to obtain in a controlled way a reduction of the pore size by around 25% and the presence of the material physical changes along the entire length of the pores. The effect of physical changes on the diffusive transport is also taken into account by determining the differences in ionic selectivity, diffusion coefficients, and electric charge by measuring the membrane potential, performed in the presence of NaCl solutions at different concentrations. A direct correlation can be obtained between the diffusion coefficients and the membrane potential.

### 2.2. Synthesis of Alumina Nanoporous Membranes

NPAMs were synthesized by two-step anodizing in oxalic acid-based electrolytes. High-purity aluminum discs (Sigma Aldrich, Steinheim, Germany), 0.5 mm thick and 25 mm in diameter, were used as matrix substrates. The aluminum discs were first cleaned by sonication in isopropanol and ethanol and then subjected to an electrolyte treatment in a mixture of perchloric acid and ethanol (1:3 vol.) at 5 °C. A voltage of 20 V was applied between the sample and a platinum counter electrode. The first anodizing process was carried out in an aqueous electrolyte of 0.3 M oxalic acid at 1–3 °C and under an anodizing potential of 40 V applied between the sample and the Pt counter electrode for 24 h. A strong stirring of the electrolyte was used during the anodizing steps to ensure the homogeneity of the electrolyte concentration, and its temperature was kept constant with an external recirculating chiller. The aluminum oxide layer raised after the first anodizing step was selectively removed by immersing the samples in an acidic solution of CrO_3_ and H_3_PO_4_ (Sigma Aldrich, Steinheim, Germany) at 35 °C for 24 h. The second anodizing step was performed under the same anodizing conditions as the first, but with a longer duration of 32 h, to adjust the final thickness of the NPAMs to around 60 nm, making them sufficiently robust to ensure their integrity during subsequent physical handling and processing.

Finally, the remaining aluminum substrate was partially removed by chemical treatment in an aqueous mixture of HCl and CuCl_2_ (Sigma Aldrich, Steinheim, Germany) by successively exposing surfaces of approximately 1 cm^2^ behind the NPAM. The alumina barrier layer that blocks filterability at the bottom of the pores was removed by additional chemical treatment in 5% aqueous H_3_PO_4_ solution at room temperature for 100 min, resulting in a two-part open porous structure for NPAM.

### 2.3. Functionalization of the Surface by Deposition of the Atomic Layer and Physical Tests

Coating with the atomic layer deposition (ALD) technique of Ox NPAMs was performed in a specialized heat reactor. Suitable precursors for the deposition of the layer of various functional oxides were selected using the deposition of the high purity atomic layer Ar as the carrier gas in all cases. NPAMs were exposed to different precursors in sequential order, using long exposure times in the range of 45–60 s, to allow gaseous precursors to diffuse through the pores of Ox NPAM samples. An extensive purge (90 s) with a high flow rate of Ar was performed between two stages of precursor insertion to remove excess unreacted gas precursor as well as reaction by-products from the ALD reaction chamber. The number of ALD cycles was adjusted according to the different growth rates of metal oxide to adjust the thickness of the deposited layer to approximately 4 nm. In our case of two-layer ALD coatings, the first layer was coated with a thickness of 4 nm, and later, the ALD precursors, as well as the deposition conditions, were conveniently modified to achieve the deposition of a second layer of Al_2_O_3_ with a thickness of 1–2 nm.

### 2.4. Materials and Methods for Membrane Biocompatibility Testing

The membrane manufactured and characterized as described above was designed to be used within an implantable device for Aβ removal from the CNS. Consequently, testing the biocompatibility of this material is mandatory before proceeding to in vivo experiments. The first essential step is to evaluate the cytotoxicity of the alumina-based membrane, determining the viability of cell cultures incubated with a culture medium after being in contact with the material in question for a certain period of time. The selected cells for this experimental model were MCF-7 and MDA-MB-231 cell lines, which are extensively used for mutagenicity and toxic studies [32].

***Cell lines***. MCF-7 (HTB-22) and MDA-MB-231 (CRM-HTB-26) cell lines purchased from the American Type Culture Collection (ATCC, Manassas, VA, USA) were maintained in DMEM (Dulbecco’s Modified Eagle Medium, Biochrom AG, Berlin, Germany), supplemented with 10% FSB (fetal bovine serum, Sigma Aldrich, Steinheim, Germany), 100 IU/mL penicillin (Biochrom AG, Berlin, Germany), and 100 µg/mL streptomycin (Biochrom AG, Berlin, Germany) at 37 °C in a humidified atmosphere of 5% CO_2_ in air. 

***MEM elution assay***. The nanoporous alumina membrane was incubated in serum-free media for 72 h at 37 °C, 5% CO_2_ atmosphere, and a humidity of 95%. After the extraction period expired, the media were supplemented with FBS (fetal bovine serum, Sigma Aldrich, Steinheim, Germany) and transferred to cell cultures. For the treatment, crude extract and dilutions of 1:1 and 1:4 were used. For dilution of the crude extract, complete media were used. 

***Evaluation of the viability of the cells was based on an MTT assay***. Briefly, cells were seeded in 96-well plates at a density of 5 × 10^3^ cells/well and allowed to attach and grow overnight. Treatment with extract was applied for 48 h using 100 µL/well of extract in different dilutions, and after the treatment period expired, the cells were washed and covered with 100 μL of fresh DMEM 10%. Then 10 μL of MTT (5 mg/mL) was added to the medium, and cells were incubated for 3 h. To determine the formed formazan, DMSO (dimethyl sulfoxide, Merck, Darmstadt, Germany) was used, and the absorbance was recorded at 570 nm. Cellular viability (%) was calculated according to the formula: % cell viability = [Absorbance]sample/[Absorbance]control × 100.

### 2.5. Optical and Structural Properties of Alumina Sample

In order to characterize the structure of the alumina sample, its X-ray diffraction pattern was recorded with a Bruker D8 Advance (Bruker AXS, Karlsruhe, Germany) X-ray diffractometer, using Cu-Kα radiation, with a wavelength of λ = 1.54056 Å.

The Raman and IR spectra of the sample were recorded with a Fourier transform (FT) Raman spectrophotometer, MultiRam model, and a Fourier transform infrared (FTIR) spectrophotometer, Vertex 80 model, respectively, both pieces of equipment being purchased from Bruker AXS, Germany.

## 3. Results and Discussion

### 3.1. Assessment of the Membrane Functionalization

**Figure 1:**
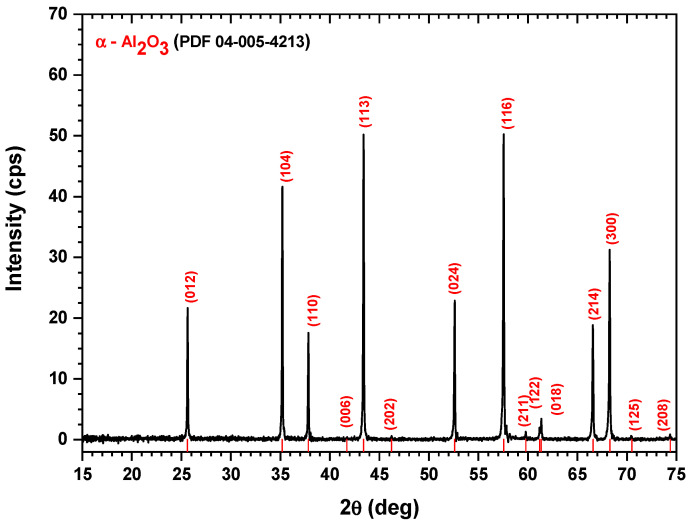
X-ray diffraction pattern of the alumina bulk sample and the diffraction lines’ positions, shown with red color, according to the PDF-04-005-4213 reference file.

Figure 1 shows the XRD diagram of the alumina sample along with the diffraction lines’ positions, shown in red color, according to the PDF-04-005-4213 reference file.

According to the XRD diffraction pattern in Figure 1, the sample showed peaks at 2θ diffraction angles equal to 25.63°, 35.19°, 37.83°, 43.36°, 43.39°, 46.24°, 52.61°, 57.56°, 59.78°, 61.16°, 61.36°, 66.57°, 68.26°, 70.49°, and 74.36°, corresponding to the crystalline planes (012), (104), (110), (006), (113), (202), (024), (116), (211), (122), (018), (214), (300), (125), and (208), respectively, of the α-Al_2_O_3_, crystallized in a rhombohedral crystal system, as revealed by the PDF-04-005-4213 reference file. These peak positions were also in good agreement with the results reported by Bajaj and Omanwar [33]. Moreover, from the XRD pattern, we may conclude that the alumina sample was very well crystallized, showing narrow and sharp XRD peaks. 

**Figure 2:**
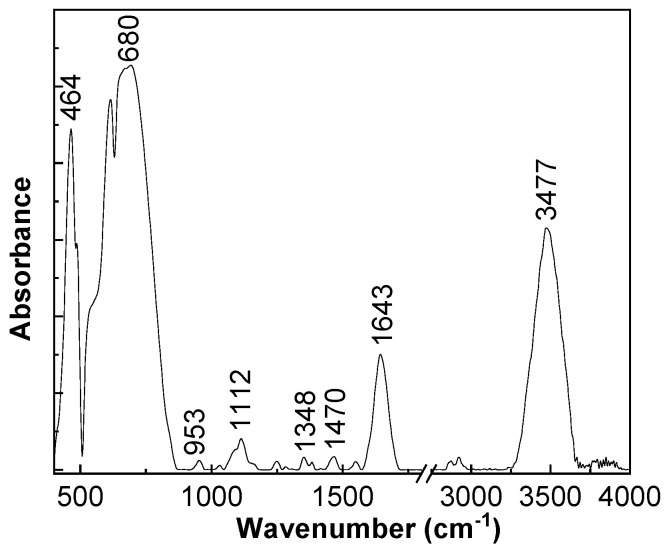
Infrared spectrum of the sample.

**Figure 3:**
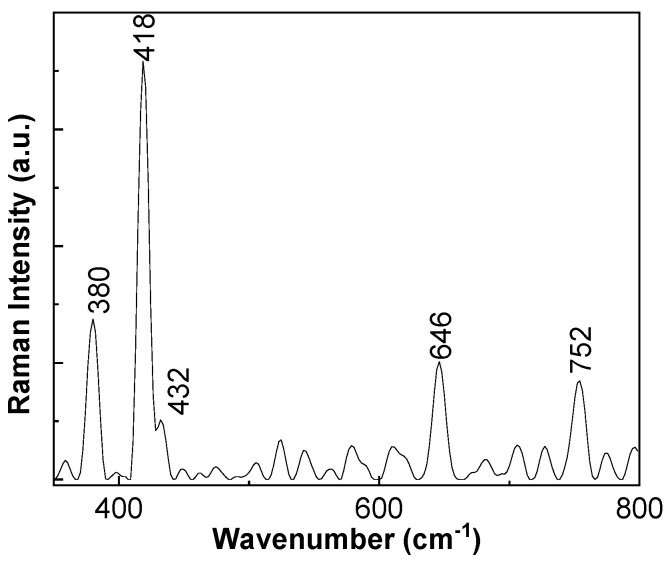
The Raman spectrum of the Ox NPAMs sample.

Figure 2 and Figure 3 show the main vibrational modes, active in the IR and Raman spectra of the M sample. Depending on the crystalline phases of Al_2_O_3_, different vibrational modes were observed [34]. In the particular case of α-Al_2_O_3_, 12 vibrational modes active in Raman spectroscopy and 10 vibrational modes active in IR spectroscopy were theoretically estimated [34].

The IR bands of the functionalized membrane, observed in Figure 2, peaked at 464–680, 953, 1112, 1348, 1470, 1643, and 3477 cm^−1^, and they were assigned to the vibrational modes of Al–O stretching in the octahedral structure, OH deformation in Al–O–H, aged α-Al_2_O_3_, Al=O, α-Al_2_O_3_, OH bending, and OH stretching, respectively [35,36,37,38]. The vibrational modes of OH bending and OH stretching indicated the presence of the water molecules adsorbed onto the α-Al_2_O_3_ surface.

The main Raman lines of the sample, highlighted in Figure 3, peaked at 380, 418, 432, 646, and 752 cm^−1^, and they were assigned to the vibrational modes E_u_, A_1g_, E_g_, A_1g_, and A_2g_ [34]. According to Liu et al., these Raman lines are characteristic of α-Al_2_O_3_ [34].

The results demonstrate the successful functionalization of the surface by deposition of the two atomic layers of Al_2_O_3_.

### 3.2. Membrane Potential Measurements

The membrane potential, or the difference in equilibrium electric potential between two electrolyte solutions of different concentrations (Cf and Cv) separated by a membrane, was measured in a test cell consisting of two glass half-cells with a magnetic stirrer at the bottom of each half-cell to minimize the effect of polarization on the membrane surfaces (e.g., with a stirring speed of 550 rpm). An Ag/AgCl reversible electrode was placed in each half-cell and connected to a digital voltmeter for cell potential measurements. These measurements were performed with different NaCl solutions (at 25 ± 2 °C, pH 5.9 ± 0.2) by maintaining a constant concentration of the solution on one side of the membrane (Cf = 0.01 M) and gradually changing the concentration of the solution on the other hand (0.002 M ≤ Cv ≤ 0.1 M). Membrane potential values (ΔΦmbr) were obtained for each pair of Cv/Cf concentrations by subtracting the electrode potential from the cell potential values, i.e., ΔΦmbr = ΔE − ΔΦelect. After measuring the membrane potential, the samples should be re-examined by SEM imaging and EDS analysis to confirm that no surface changes are due to the slightly acidic pH of the test solutions.

**Figure 4:**
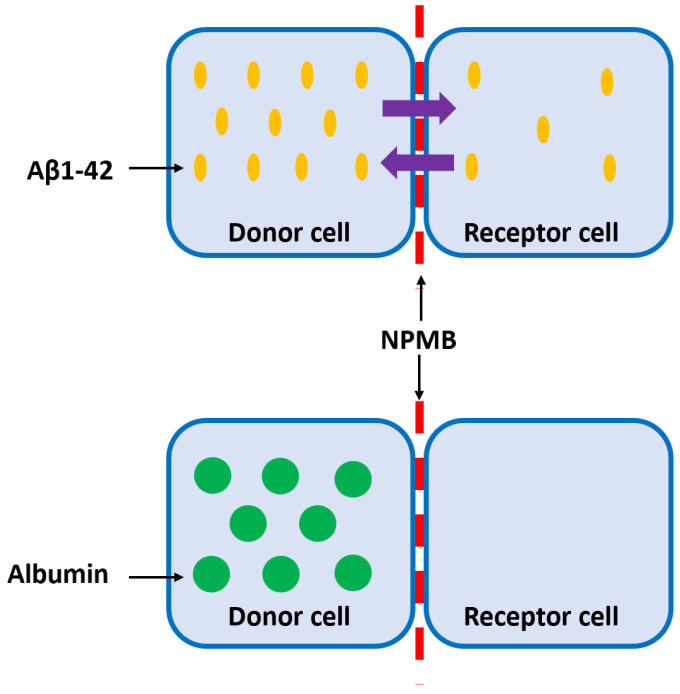
Schematic representation of the in vitro selective permeability study of the nanoporous membrane (NPMB): while there is a bidirectional diffusion (purple arrows) of Aβ1-42 (yellow dots) between the donor and the receptor cells, no diffusion is observed in the case of albumin (green dots).

The Aβ peptide is one of the main biomarkers of AD. Normally, this protein is soluble in low concentrations but tends to self-aggregate, forming increasingly complex and insoluble structures that give rise to characteristic extracellular plaques. Soluble Aβ is found in a constant balance between CSF and cerebral parenchyma. The new therapeutic strategy is based on removing Aβ from the CSF, shifting the balance, and decreasing the concentration of Aβ in the brain parenchyma without releasing therapeutic agents into the parenchyma. To achieve this, customized nanomembranes must be designed, and the permeability of these nanomembranes to Aβ must be evaluated, respectively, in terms of specific therapeutic agents associated with Aβ elimination, such as albumin. In order to determine the selective permeability of the nanomembrane to monomers and oligomers of type Aβ1-42 (the most toxic form of Aβ), but not to albumin, experiments were designed, as shown in Figure 4. First, the tests evaluated whether the Aβ1-42 peptide diffuses from the donor to the recipient cell and vice versa; subsequently, the therapeutic agent (albumin) did not diffuse from the receptor to the donor cell, and vice versa, demonstrating the impermeability of the membrane. In the case of Aβ1-42, the system should be left for a maximum of 72 h and Aβ1-42 levels to be determined by ELISA. For albumin tests, its levels must be analyzed for 72 to 196 h.

Nanoporous membranes are attached to a permeation chamber that separates two cells: the donor and the receptor. Thus, both compartments are in contact but separated by the nanoporous membrane.

To achieve this, adapted functional nanoporous membranes with pore sizes on the deca-nanometric scale were designed to eliminate Aβ1-42.

For nanoporous membranes, the following preliminary characteristics related to Aβ size were considered:

- The maximum therapeutic surface, in the shape of a circle with a diameter of 10 mm;
- Thickness: 50–60 μm;
- Pore size: 10.00 nm;
- Maximum dispersion of pore size: 0.50 nm;
- Interpore space: from 30 to 100 nm.

The supraphysiological levels of human peptide (20 pg/mL) and albumin (2 mg/mL), diluted in artificial CSF, were added to the donor or recipient cells. Samples from both cells were taken at different times, and Aβ1-42 and albumin levels were analyzed by ELISA, as described in previous studies [39,40,41,42,43].

Preliminary tests, conducted after 72 h, showed that the albumin levels did not change, remaining in the original cell, but after 72 h, the concentration of Aβ1-42 peptide was similar in both cells, regardless of the concentration in the source cell. Moreover, even after a time of 192 h, the albumin levels remained at a very low level, which confirms that the preliminary dimensions of the membranes were judiciously determined, and it was possible to proceed to the elaboration of the membranes in the laboratory.

### 3.3. Assessment of the Membrane Biocompatibility on Cell Cultures

Membrane biocompatibility was investigated in vitro on two distinct cell lines (MCF-7 and MDA-MB-231) treated with different dilutions (25% and 50%) of concentrated extract 48 h after treatment was applied. At that time point, the extract did not show any decrease in the viability of the cell cultures; however, the viability of the treated wells was a little higher than in the case of the control group. In addition, by comparing the treated groups, no differences between groups were registered. Thus, in this preliminary test for assessment of biocompatibility, the tested membrane proved to be fully compatible with the cell cultures.

Assessment of the biocompatibility of the alumina membrane was based on the quantification of live and dead cells by MTT assay, using incubated media with the membrane. Contact time between media and alumina membrane was 72 h, and prior to addition to cell cultures was supplemented with 10% FBS. For the cell treatment, crude extract and 2 dilutions (1:2 and 1:4) starting from the crude extract obtained by incubation of alumina membrane in media for 72 h were used. To dilute the extract, complete media were used, and aliquots from crude extract were combined with fresh media. Cells in the control group were assumed to have 100% viability and were used as a reference for the calculation of viability in the test groups. 

**Figure 5:**
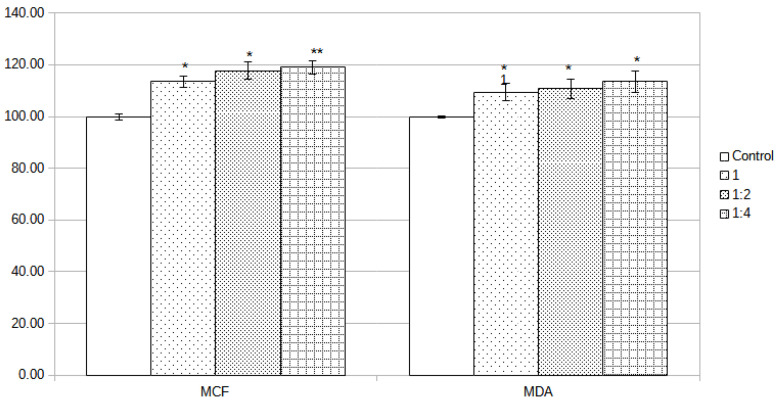
Viability of the MCF-7 and MDA-MB-231 cell lines as determined by MTT assay after 48 h of incubation with the tested extract in different dilutions: 1—concentrated extract, 1:2—1 part extract and 1 part DMEM, 1:4—1 part extract and 3 parts DMEM. The extract was obtained by incubation in serum-free DMEM for 72 h at 37 °C in a 5% CO_2_ atmosphere and 95% humidity. The control group received only normal media. Data are mean and SEM values (n  = 6). Statistical significance was obtained by Student *t*-tests, comparing every treated group with the control group. * denotes *p* < 0.05; ** denotes *p* < 0.01.

The alumina membrane did not influence the viability of MCF-7 cells, the only noticeable effect being an increase in the number of cells (viable cells). The crude extract determined an increase in cell number/viability with 13.58%, dilution 1:2 with 17.74%, and dilution 1:4 with 19.03%. In addition, in the case of MDA-MB-231 cells, the alumina membrane extract did not influence the viability of the cells; however, an increase in the number of alive cells was registered. The amplitude was lower than in the case of MDA-MB-231 cells but still was over the 100% viability of the control group with 9.45% (crude extract), 10.71% (1:2 dilution), and 13.52% (1:4 dilution). The calculated statistical significance by Student’s t-test revealed that all differences in cell viability were statistically relevant, as can be observed from Figure 5 (* ≤0.05, ** ≤0.01). The detailed numbers can be observed in Table 2. 

Unprecedented progress of nanotechnologies in the area of biomedicine has led to breakthroughs in theranostics and multiple new applications in different diseases. In addition to the utility of nanotechnology in different medical fields, concerns about safety and the biocompatibility of the newly developed devices based on nanoscale components have arisen.

Many biomedical applications are using nanoporous materials in devices for immune isolation, dialysis, and bioanalysis or for drug delivery and design of biosensors [44]. In addition, cell separation and sorting, micro-arrangement, in-vitro tissue reconstruction, high-throughput manipulation and analysis, and real-time sensing are of interest. Membranes used in this kind of application should meet some key features, such as pore size of a few nanometers or less, high selectivity and porosity, low thickness, and mechanical and chemical stability [45,46].

Inorganic membranes present some advantages over polymeric membranes: increased chemical and mechanical stability, and better control of the pore size and pore size distribution by varying the fabrication parameters [47,48,49]. Another advantage of the inorganic membranes is the possibility of chemically modification to repel the adhesion of blood proteins and immune cells that may biofoul the pores and reduce the diffusion of therapeutic agents or the efficiency of filtration [50,51].

The use of semi-permeable barriers for immune isolation is a promising approach for improving treatment for AD and Parkinson’s disease, providing new ways to control the evolution of neurodegenerative diseases. Nanoporous alumina biocapsules were developed as immune isolation devices and support the viability and functionality of encapsulated *β* cells [52].

One of the major problems with new materials and their use in biomedical applications is the compatibility with the host, as some reactions from the host, such as foreign body reactions and complement activation, could occur, making them unusable or inefficient [52].

This part of our study focused on evaluating the biocompatibility of a newly developed nanoporous alumina membrane for selective filtration of Aβ related to AD. In vitro tests, using the extract method and MTT assay for viability recording, did not indicate any cytotoxic effects as measured on two cell lines. Those results are in accordance with other studies that evaluated the biocompatibility of alumina membranes [53,54,55].

Biocompatibility of the tested nanoporous alumina membrane offers the possibility to design and integrate the membrane into implantable or wearable biomedical devices to address the treatment of different diseases. However, the principal challenges in the development of new nanoporous supports reside in their in vivo biocompatibility and capacity to avoid biofouling due to contact with biological structures. Future developments will rely on inorganic membranes functionalized to act as intelligent devices with adaptative traits that will permit better integration in the complex physiology of living organisms.

## 4. Conclusions and Future Research Directions

As neurodegenerative diseases, especially AD, are expected to pose increasing burdens at both individual and socioeconomic levels, and in the absence of currently available curative treatments, there is a need to find new effective therapeutic approaches. Based on the Aβ hypothesis, we propose a novel therapeutic direction concerning the filtration of Aβ from the CSF; in this regard, designing a functionalized nanoporous membrane as the main part of a filtrating experimental model that will be used in further in vivo studies.

Firstly, our work described the complex process of nanoporous membrane development and functionalization. Studies using X-ray diffraction, IR spectroscopy, and Raman scattering have demonstrated that the synthesis of nanoporous membrane involves the generation of α-Al_2_O_3_. Subsequently, via the MEM elution assay and MTT assay and by using two different cell lines (MCF-7 and MDA-MB-231), the biocompatibility of the functionalized nanoporous membrane was demonstrated. 

Our preliminary results are encouraging, suggesting that the proposed experimental model is safe for in vivo testing. In this context, an interesting option would be the development of an experimental device endowed with the abovementioned nanoporous membrane, capable of clearing macromolecules, including normal and pathological proteins, from different body fluids. Via this membrane, selective and continuous apheresis of blood and/or CSF should be possible. In addition to the apheresis module, the prototype could also include additional components that enable access to the CSF and the possibility to infuse therapeutic agents at that level. Initial in vivo experiments might be conducted on AD animal models, with transgenic mice and rats being the most appropriate. Finally, the impact of the proposed device on AD evolution could be assessed by measuring the Aβ load at the CSF and brain levels. This study opens an interesting research direction based on the therapeutic impact of Aβ clearance from the CSF and represents the first step in our more elaborate research regarding the development of an implantable device endowed with the described nanoporous membranes for the effective use of anti-AD therapeutics.

## Figures and Tables

**Table 1:** Classification of membranes according to pore type and applications.

Type of Membrane	Pore Size	Application	Filtrated Species/Molecules
Macroporous membrane	>50 nm	Microfiltration Ultrafiltration	Oily emulsions Bacteria
Mesoporous membrane	2–50 nm	Ultrafiltration	Viruses
Supermicroporous membrane	<2 nm (>7 Å)	Nanofiltration	Antibiotics Ions Small neutral molecules (gas and liquid phase)
Ultramicropores membrane	<7 Å	Pervaporation Vapor permeation Gas separation	

Abbreviations used in Table 1: nm—nanometer; Å—angstrom.

**Table 2:** Data table regarding the viability of the MCF-7 and MDA-MB-231 cell lines as determined by MTT assay after 48 h of incubation with the tested extract in different dilutions (1; 1:2; 1:4).

	MCF		MDA	
	Mean ± SEM	*p* Value	Mean ± SEM	*p* Value
Control	100.00 ± 1.16		100.00 ± 0.38	
1	113.58 ± 2.17	<0.05	109.45 ± 3.30	<0.05
1:2	117.74 ± 3.31	<0.05	110.71 ± 3.91	<0.05
1:4	119.03 ± 2.59	<0.01	113.52 ± 4.15	<0.05

## Data Availability

The data that support the findings of this study are available upon request from the corresponding author.
